# Crystal structure of 5-(β-d-gluco­pyran­osyl­thio)-*N*-(4-methyl­phen­yl)-1,3,4-thia­diazol-2-amine

**DOI:** 10.1107/S2056989023005248

**Published:** 2023-06-20

**Authors:** Mamdouh A. Abu-Zaied, Ali M. S. Hebishy, Galal H. Elgemeie, Hagar T. Salama, Peter G. Jones

**Affiliations:** a Green Chemistry Department, National Research Centre, Dokki, Giza, Egypt; b Chemistry Department, Faculty of Science, Helwan University, Cairo, Egypt; c Institut für Anorganische und Analytische Chemie, Technische Universität Braunschweig, Hagenring 30, D-38106 Braunschweig, Germany; Universität Greifswald, Germany

**Keywords:** glucose, thia­diazole, crystal structure, hydrogen bonds

## Abstract

In the structure of the title compound, C_15_H_19_N_3_O_5_S_2_, the bond lengths at the linking sulfur atom are significantly different [1.7473 (17) and 1.811 (2) Å], and the angle at the exocyclic nitro­gen atom is wide at 128.45 (18)°. The inter­planar angle between the tolyl and thia­diazole rings is 9.2 (1)°. The complex hydrogen-bonding pattern, involving five donors and five acceptors, can be broken down into a one-dimensional ribbon parallel to the *b* axis, involving hydrogen bonds of the sugar residues only, and a two-dimensional layer structure parallel to the *ab* plane, based on the N—H⋯O and O—H⋯N hydrogen bonds.

## Abstract 2

In the title compound, the angle between the tolyl and thia­diazole rings is 9.2 (1)°. The hydrogen bonding is a combination of a ribbon involving hydrogen bonds of the sugar residues, and a layer based on N—H⋯O and O—H⋯N hydrogen bonds.

## 1. Chemical context

There has been considerable recent inter­est in the chemistry of compounds involving both heterocyclic and carbohydrate moieties (Lopes *et al.*, 2021 ▸). Heterocyclic thio­glycosides are promising candidates in synthetic carbohydrate research, and some of these compounds have displayed various antagonistic activities (Abu-Zaied *et al.*, 2011 ▸, 2019 ▸; Khedr *et al.*, 2022 ▸). 1,3,4-Thia­diazo­les are an important class of heterocycles that have found diverse applications in organic synthesis, biological applications, and pharmaceuticals (Sun *et al.*, 2011 ▸), thus motivating researchers to prepare many derivatives of these compounds (Matysiak, 2015 ▸). Our inter­est in synthesizing novel active heterocycles (Khedr *et al.*, 2022 ▸; Hebishy *et al.*, 2022 ▸; Abdallah *et al.*, 2022 ▸) and their glycosylic derivatives (Azzam *et al.*, 2022*a* ▸,*b* ▸) led us to expect that 1,3,4-thia­zole compounds and their sugar-linked products could be valuable systems for designing novel cytotoxic agents (Yang *et al.*, 2012 ▸). In our previous work, many anti­viral heterocyclic thio­glycosides, such as azole and azine thio­glycosides, were synthesized and found to display effective cytotoxicities (Elgemeie *et al.*, 2016 ▸, 2017*a* ▸,*b* ▸, 2018 ▸; Elgemeie & Mohamed-Ezzat, 2022*a* ▸,*b* ▸). We have also reported that di­hydro­pyridine thio­glycosides can be used as inhibitors of the glycosyl­ation of proteins (Scala *et al.*, 1997 ▸).

**Figure 1:**
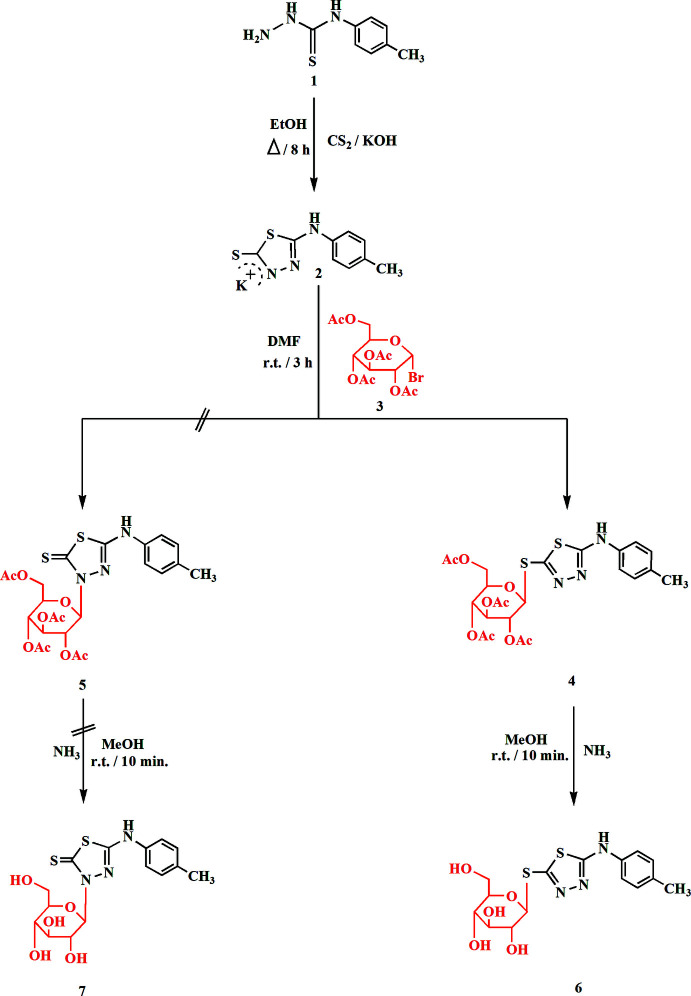
Reaction scheme for the synthesis of **6**.

In the current study, we have designed a facile synthesis of 1,3,4-thia­diazole thio­glucosides by coupling of potassium 1,3,4-thia­diazo­lates and protected α-d-gluco­pyranosyl bromide. Our target derivative was synthesized by the reaction of the thio­semicarbazide derivative **1** with carbon di­sulfide in boiling KOH/EtOH to afford the corresponding potassium 1,3,4-thia­diazole thiol­ate **2** in good yield (Fig. 1 ▸). Compound **2** was then coupled with acetyl­ated α-d-gluco­pyran­ose bromide **3** in DMF at room temperature to give a product that could in principle be either the 1,3,4-thia­diazole *S*-glucoside **4** or the isomeric *N*-glucoside **5**, corresponding to two different modes of glycosyl­ation. Deprotection then provided a final product that should be either the 1,3,4-thia­diazole *S*-glucoside **6** or the isomeric *N*-glucoside **7**. Spectroscopic data cannot distinguish these two structures with absolute certainty, although it had already been proposed that a simple S_N^2^_ reaction between **2** and **3** would give the *β*-glucoside product **4** (Masoud *et al.*, 2017 ▸; Hammad *et al.*, 2018 ▸), which would imply the final formation of **6**. 
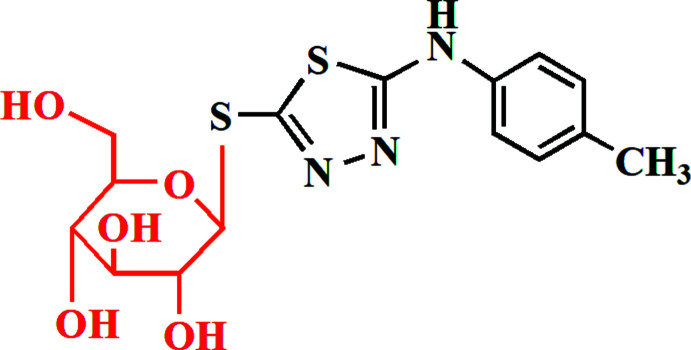


This is consistent with the spectroscopic data; thus the ^1^H NMR spectrum of **6** showed the signal of the anomeric proton as a doublet at *δ* 4.72 (*J*_1‘,2‘_ = 10.8 Hz), strongly implying a *β*-d-configuration. The ^13^C NMR spectrum exhibited a signal at *δ* 86.89 corresponding to C-1′, whereas the signals at *δ* 61.34, 70.00, 73.07 and 78.32, 81.42 were allocated to C-6′, C-4‘, C-2′, C-3′ and C-5′. The X-ray structure determination, presented here, unambiguously shows the isolated product to be the 1,3,4-thia­diazole-5-thio­glucoside **6** (Fig. 1 ▸).

## 2. Structural commentary

**Figure 2:**
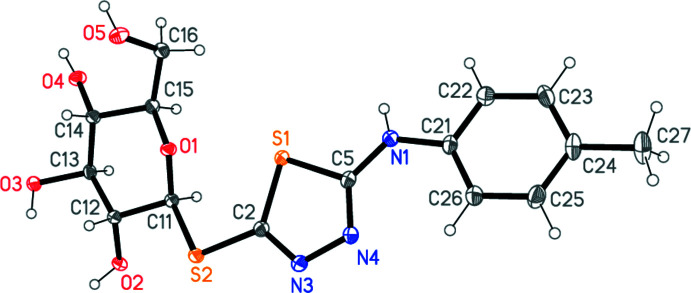
The mol­ecule of compound **6** in the crystal. Ellipsoids represent 50% probability levels.

The mol­ecular structure of compound **6** is shown in Fig. 2 ▸. Note that the standard sugar numbering has been slightly modified (to C11–16) for the crystallographic numbering. Mol­ecular dimensions (Table 1 ▸) may be regarded as normal; *e.g*. the bond lengths at S2 are significantly different, consistent with the different hybridization of the carbon atoms [C2—S2 = 1.7473 (17), C11—S2 = 1.811 (2) Å], and the angle C5—N1—C21 is wide at 128.45 (18)°. The inter­planar angle between the tolyl and thia­diazole rings is 9.2 (1)°. The β (equatorial) position of the substituent at the glucose ring is confirmed by the torsion angle C15—O1—C11—S2 of 177.11 (10)°. The absolute configuration was confirmed by the Flack parameter, with chiralities *S*,*R*,*S*,*S*,*R* at C11–15 respectively consistent with the presence of d-glucose.

## 3. Supra­molecular features

**Figure 3:**
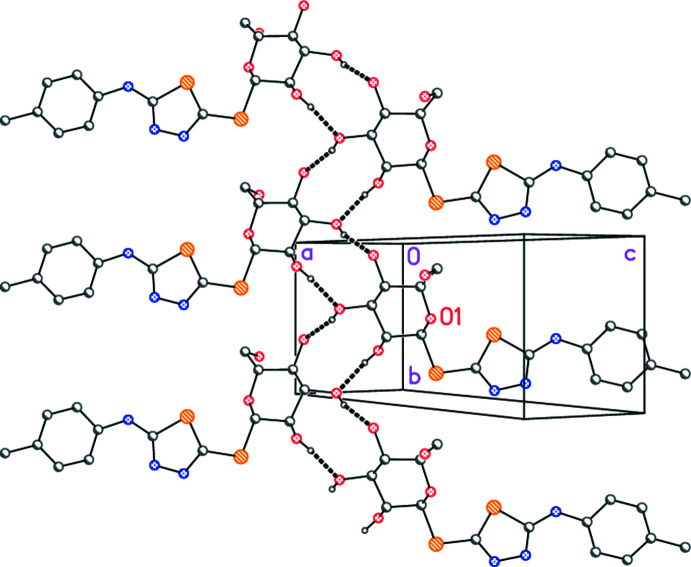
Packing diagram of compound **6**: the glucose-based substructure involving two O—H⋯O hydrogen bonds (indicated by thick dashed lines). The view direction is perpendicular to the plane (105). The labelled atom (O1) indicates the asymmetric unit.

With five classical hydrogen bonds (Table 2 ▸), the mol­ecular packing of **6** might be expected to be three-dimensional and complex, and this is indeed the case. However, the packing may be analysed in terms of more easily assimilable substructures. One, formally one-dimensional, substructure involving the sugar residues can readily be identified (Fig. 3 ▸), the hydrogen bonds O2—H02⋯O3 and O3—H03⋯O4(−*x* + 3, *y* + 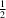 , −*z* + 1 for both) combine *via* the 2_1_ screw axis to form ribbons of mol­ecules parallel to the *b* axis. The ribbons lie in layers roughly parallel to (105). The OH group at C16 is directed away from its layer to form contacts to the neighbouring layer.

**Figure 4:**
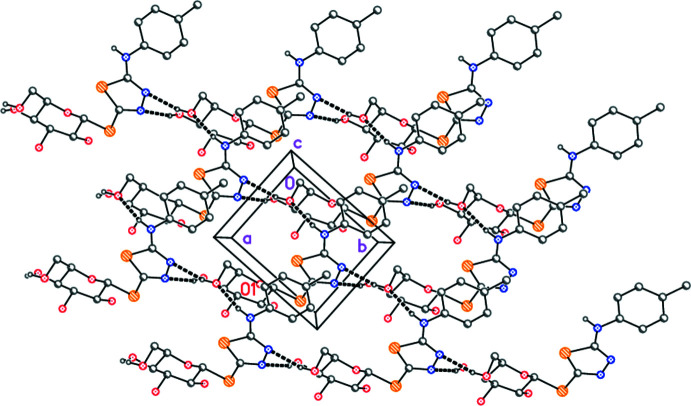
Packing diagram of compound **6**: the layer substructure involving the O—H⋯N and N—H⋯O hydrogen bonds (indicated by thick dashed lines). The view direction is perpendicular to the *ab* plane. The labelled atom (O1) indicates the asymmetric unit.

A second, two-dimensional, substructure (Fig. 4 ▸) is based on the remaining three hydrogen bonds (of the types O—H⋯N and N—H⋯O), and connects the mol­ecules first by translation (both O—H⋯N hydrogen bonds; *x* + 1, *y* − 1, *z*) to form chains parallel to (1 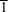 0) (horizontal in the Figure), and secondly by *a-*axis translation (the N—H⋯O hydrogen bond; *x* − 1, *y*, *z*). The overall effect is to create layers parallel to the *ab* plane.

The contact O5—H05⋯N3(*x* + 1, *y* − 1, *z*) may be regarded as the second, weaker, branch of a three-centre inter­action, but this contact is omitted from the packing diagrams for clarity. Similarly, the two C—H⋯S inter­actions are probably inter­pretable as ‘weak’ hydrogen bonds, but we do not discuss their structural role in detail.

## 4. Database survey

The search employed the routine ConQuest (Bruno *et al.*, 2002 ▸), part of Version 2022.3.0 of the Cambridge Structural Database (Groom *et al.*, 2016 ▸). We searched for pyran­ose sugars attached by a sulfur atom to heterocycles containing more than one heteroatom (a larger subset of hits was edited by hand). The six structures thus found were derivatives of 1,2,4-triazole (refcode HEKPUL; El Ashry *et al.*, 2018 ▸), 1,3,4-oxa­diazole (IZAJEY; Qiu & Xu, 2004 ▸), benzoxazole (JIPYUD and JIPZAK; Kamat *et al.*, 2007 ▸), 1,3,5-oxa­thia­zole, involving a spiro junction at the sugar C1 atom (PIWVIA; Praly *et al.*, 1994 ▸) and 1,3,4-thia­diazole (SASXIU; Qiu *et al.*, 2005 ▸). In all except PIWVIA, the sugar OH groups were substituted with ester functions. The structure SASXIU, despite having the same heterocycle as **6**, (but with a 2-phenyl substituent), has a markedly different relative orientation of the glucose and heterocyclic rings, with a torsion angle C_gluc_—S_gluc_—C_hetero_—S_hetero_ of 78.10 (10)° compared to the value of 18.65 (13)° for **6**.

## 5. Synthesis and crystallization

Preparation of inter­mediate **4**: A solution of 2,3,4,6-tetra-*O-*acetyl-α-d-gluco­pyranosyl bromide (**3**) (10 mmol) in dry DMF (15 mL) was added dropwise over 30 min to a solution of the potassium thiol­ate **2** (10 mmol) in 20 mL of DMF. The reaction mixture was stirred at room temperature until completion (monitored by TLC), then the mixture was poured into ice–water, and the resulting precipitate was collected by filtration, dried, and crystallized from ethanol to give the acetyl­ated glucoside **4**.

N·B.: The NMR data, as given here and in Section 1, refer to sugar numbering C1′–C6′, which is different from the crystallographic numbering of the glucose moiety in **6** (C11–C16).

White powder (EtOH); yield 93%; m.p. 479–481 K; IR (cm^−1^): υ 3360 (NH), 2949 (CH_3_), 1741 (C=O); ^1^H NMR (400 MHz, DMSO-*d*_6_): δ 1.91–2.05 (4*s*, 12H, 4 × OAc), 2.27 (*s*, 3H, CH_3_), 4.07–4.19 (*m*, 3H, H-6′, H-6′′, H-5′), 4.90–4.98 (*m*, 2H, H-4′, H-2′), 5.39 (*t*-like, 1H, *J* = 10.8 Hz, H-3′), 5.40 (*d*, 1H, *J*_1′–2′_ = 7.1 Hz, H-1′), 7.16 (*d*, 2H, *J* = 8.0 Hz, Ar-H), 7.46 (*d*, 2H, *J* = 7.2 Hz, Ar-H), 10.45 (*s*, D_2_O exchangeable, 1H, NH); ^13^C NMR (100 MHz, DMSO-*d_6_*): δ 20.70, 20.81, 20.90 (5 × CH_3_), 62.20 (C-6′), 68.23 (C-4′), 70.04 (C-2′), 73.14 (C-3′), 75.17 (C-5′), 82.99 (C-1′), 118.35 (2C, Ar-C), 130.05 (2C, Ar-C), 131.99 (Ar-C), 138.30 (Ar-C), 145.21 (C-2), 167.88 (C-5), 169.50, 169.74, 169.98, 170.48 (4C=O). Analysis calculated for C_23_H_27_N_3_O_9_S_2_ (553.61): C 49.90, H 4.92, N 7.59, S 11.58. Found: C 49.82, H 4.81, N 7.52, S 11.46%.

Preparation of title compound **6**: In a 50 mL flask, the tetra­acetyl­ated glucoside derivative **4** (0.01 mol) was dissolved in 20 mL of dry methanol, and then ammonia gas was passed through the solution at 273 K for 10 min. The mixture was then stirred until the reaction was complete (monitored by TLC using chloro­form/methanol 9:1). The solution was concentrated under reduced pressure to afford a solid residue, which was washed several times with boiling chloro­form. The residue was dried, purified and recrystallized from ethanol to give the corresponding free glucoside **6**.

Colourless crystals (EtOH); yield 62%; m.p. 472–474 K; IR (cm^−1^): ν 3271 (OH), 2921 (CH); ^1^H NMR (400 MHz, DMSO-*d*_6_): δ 2.25 (*s*, 3H, CH_3_), 3.11–3.22 (*m*, 2H, H-6′, H-6′′), 3.23–3.29 (*m*, 2H, H-5′, H-4′), 3.49–3.56 (*m*, 1H, H-3′), 3.71–3.76 (*m*, 1H, H-2′), 4.59 (*t*, 1H, *J*_OH–H-6′′_ = 3.6 Hz, D_2_O-exchangeable, 6′′-OH), 4.72 (*d*, 1H, *J*_1′-2′_ = 10.8 Hz, H-1′), 5.05 (d, 1H, *J* = 6.4 Hz, D_2_O-exchangeable, OH), 5.17 (*d*, 1H, *J* = 6.4 Hz, D_2_O-exchangeable, OH), 5.53 (*d*, 1H, *J* = 8.0 Hz, D_2_O-exchangeable, OH), 7.14 (*d*, 2H, *J* = 11.2 Hz, Ar-H), 7.47 (*d*, 2H, *J* = 12.4 Hz, Ar-H), 10.32 (*s*, D_2_O exch., 1H, NH); ^13^C NMR (100 MHz, DMSO-*d*_6_): δ 20.82 (CH_3_), 61.34 (C-6′), 70.00 (C-4′), 73.07 (C-2′), 78.32 (C-3′), 81.42 (C-5′), 86.89 (C-1′), 117.98 (2C, Ar-C), 129.96 (2C, Ar-C), 131.33 (Ar-C), 138.50 (Ar-C), 150.03 (C-2), 166.90 (C-5). Analysis calculated for C_15_H_19_N_3_O_5_S_2_ (385.08): C 46.75, H 4.94, N 10.91, S 16.62. Found: C 46.6, H 4.8, N 10.9, S 16.5%.

## 6. Refinement

Crystal data, data collection and structure refinement details are summarized in Table 3 ▸. Hydrogen atoms of the NH and OH groups were refined freely, the latter however with O—H distances restrained to be approximately equal (command SADI). The methyl group was included as an idealized rigid group allowed to rotate but not tip (C—H = 0.98 Å, H—C—H = 109.5°). Other hydrogen atoms were included using a riding model starting from calculated positions (C—H_aromatic_ 0.95 Å, C—H_methine_ 1.00 Å, C—H_methyl­ene_ 0.99 Å). The *U*(H) values were fixed at 1.5 × *U*_eq_ of the parent carbon atoms for the methyl group and 1.2 × *U*_eq_ for other hydrogens. An extinction correction was performed; the extinction parameter as defined by Sheldrick (2015*a* ▸) refined to 0.0009 (3). The absolute configuration (corresponding to d-glucose) was confirmed by the Flack parameter of −0.006 (5).

## Supplementary Material

Crystal structure: contains datablock(s) I, global. DOI: 10.1107/S2056989023005248/yz2035sup1.cif

Structure factors: contains datablock(s) I. DOI: 10.1107/S2056989023005248/yz2035Isup2.hkl

Supporting information file. DOI: 10.1107/S2056989023005248/yz2035Isup3.cml

CCDC reference: 2269285

Additional supporting information:  crystallographic information; 3D view; checkCIF report

## Figures and Tables

**Table 1:** Selected geometric parameters (Å, °)

S1—C2	1.7322 (19)	N4—C5	1.309 (3)
S1—C5	1.7525 (18)	C5—N1	1.358 (2)
C2—N3	1.298 (2)	C11—S2	1.811 (2)
C2—S2	1.7473 (17)	N1—C21	1.411 (2)
N3—N4	1.397 (2)		
			
C2—S1—C5	86.59 (9)	C5—N4—N3	111.44 (15)
N3—C2—S1	114.31 (13)	N4—C5—N1	128.67 (17)
N3—C2—S2	119.74 (14)	N4—C5—S1	114.15 (14)
S1—C2—S2	125.88 (10)	N1—C5—S1	117.17 (15)
C2—N3—N4	113.29 (16)	C5—N1—C21	128.45 (18)
			
S2—C11—O1—C15	177.11 (10)	O1—C11—S2—C2	−55.86 (12)
N3—C2—S2—C11	−164.51 (14)	C12—C11—S2—C2	−174.12 (11)
S1—C2—S2—C11	18.65 (13)		

**Table 2:** Hydrogen-bond geometry (Å, °)

*D*—H⋯*A*	*D*—H	H⋯*A*	*D*⋯*A*	*D*—H⋯*A*
O2—H02⋯O3^i^	0.82 (2)	1.87 (2)	2.6846 (19)	174 (3)
O3—H03⋯O4^i^	0.84 (2)	1.93 (2)	2.7375 (18)	161 (3)
O4—H04⋯N3^ii^	0.81 (2)	2.06 (2)	2.848 (2)	162 (3)
O5—H05⋯N4^ii^	0.83 (2)	2.11 (2)	2.899 (2)	159 (3)
O5—H05⋯N3^ii^	0.83 (2)	2.60 (2)	3.282 (2)	141 (2)
N1—H01⋯O5^iii^	0.84 (3)	2.05 (3)	2.853 (2)	158 (2)
C14—H14⋯S1^iv^	1.00	2.74	3.7044 (18)	163
C16—H16*B*⋯S2^v^	0.99	2.71	3.570 (2)	145

Symmetry codes: (i) 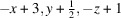 ; (ii) 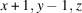 ; (iii) 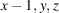 ; (iv) 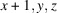 ; (v) 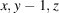 .

**Table 3:** Experimental details

Crystal data	
Chemical formula	C_15_H_19_N_3_O_5_S_2_
*M*_r_	385.45
Crystal system, space group	Monoclinic, *P*2_1_
Temperature (K)	100
*a*, *b*, *c* (Å)	6.23840 (6), 7.4355 (1), 18.32032 (17)
β (°)	91.4183 (8)
*V* (Å^3^)	849.54 (2)
*Z*	2
Radiation type	Cu *K*α
μ (mm^−1^)	3.14
Crystal size (mm)	0.10 × 0.08 × 0.02

Data collection	
Diffractometer	XtaLAB Synergy
Absorption correction	Multi-scan (*CrysAlis PRO*; Rigaku OD, 2021 ▸)
*T*_min_, *T*_max_	0.851, 1.000
No. of measured, independent and observed [*I* > 2σ(*I*)] reflections	70354, 3520, 3502
*R*_int_	0.030
(sin θ/λ)_max_ (Å^−1^)	0.633

Refinement	
*R*[*F* ^2^ > 2σ(*F* ^2^)], *wR*(*F* ^2^), *S*	0.019, 0.050, 1.03
No. of reflections	3520
No. of parameters	248
No. of restraints	7
H-atom treatment	H atoms treated by a mixture of independent and constrained refinement
Δρ_max_, Δρ_min_ (e Å^−3^)	0.30, −0.21
Absolute structure	Flack *x* determined using 1554 quotients [(*I* ^+^)−(*I* ^−^)]/[(*I* ^+^)+(*I* ^−^)] (Parsons *et al.*, 2013 ▸)
Absolute structure parameter	−0.006 (5)

Computer programs: *CrysAlis PRO* (Rigaku OD, 2021 ▸), *SHELXT* (Sheldrick, 2015*b* ▸), *SHELXL2018/3* (Sheldrick, 2015*a* ▸) and *XP* (Siemens, 1994 ▸).

## supplementary crystallographic information

### Crystal data

**Table:**

C_15_H_19_N_3_O_5_S_2_	*F*(000) = 404
*M**_r_* = 385.45	*D*_x_ = 1.507 Mg m^−^^3^
Monoclinic, *P*2_1_	Cu *K*α radiation, λ = 1.54184 Å
*a* = 6.23840 (6) Å	Cell parameters from 61296 reflections
*b* = 7.4355 (1) Å	θ = 2.4–77.5°
*c* = 18.32032 (17) Å	µ = 3.14 mm^−^^1^
β = 91.4183 (8)°	*T* = 100 K
*V* = 849.54 (2) Å^3^	Plate, colourless
*Z* = 2	0.10 × 0.08 × 0.02 mm

### Data collection

**Table:**

XtaLAB Synergy diffractometer	3520 independent reflections
Radiation source: micro-focus sealed X-ray tube, PhotonJet (Cu) X-ray Source	3502 reflections with *I* > 2σ(*I*)
Mirror monochromator	*R*_int_ = 0.030
Detector resolution: 10.0000 pixels mm^-1^	θ_max_ = 77.3°, θ_min_ = 2.4°
ω scans	*h* = −7→7
Absorption correction: multi-scan (CrysAlisPro; Rigaku OD, 2021)	*k* = −9→8
*T*_min_ = 0.851, *T*_max_ = 1.000	*l* = −23→23
70354 measured reflections	

### Refinement

**Table:**

Refinement on *F*^2^	H atoms treated by a mixture of independent and constrained refinement
Least-squares matrix: full	*w* = 1/[σ^2^(*F*_o_^2^) + (0.0289*P*)^2^ + 0.2573*P*] where *P* = (*F*_o_^2^ + 2*F*_c_^2^)/3
*R*[*F*^2^ > 2σ(*F*^2^)] = 0.019	(Δ/σ)_max_ < 0.001
*wR*(*F*^2^) = 0.050	Δρ_max_ = 0.30 e Å^−^^3^
*S* = 1.03	Δρ_min_ = −0.21 e Å^−^^3^
3520 reflections	Extinction correction: SHELXL-2018/3 (Sheldrick, 2015a), Fc^*^=kFc[1+0.001xFc^2^λ^3^/sin(2θ)]^-1/4^
248 parameters	Extinction coefficient: 0.0009 (3)
7 restraints	Absolute structure: Flack *x* determined using 1554 quotients [(*I*^+^)-(*I*^-^)]/[(*I*^+^)+(*I*^-^)] (Parsons *et al.*, 2013)
Primary atom site location: dual	Absolute structure parameter: −0.006 (5)
Hydrogen site location: mixed	

### Special details

**Table:**

Geometry. All esds (except the esd in the dihedral angle between two l.s. planes) are estimated using the full covariance matrix. The cell esds are taken into account individually in the estimation of esds in distances, angles and torsion angles; correlations between esds in cell parameters are only used when they are defined by crystal symmetry. An approximate (isotropic) treatment of cell esds is used for estimating esds involving l.s. planes. Least-squares planes (x,y,z in crystal coordinates) and deviations from them (* indicates atom used to define plane) 3.7080 (0.0039) x + 2.0545 (0.0042) y + 13.5622 (0.0105) z = 13.7691 (0.0045) * 0.0205 (0.0007) S1 * -0.0130 (0.0010) C2 * -0.0030 (0.0011) N3 * 0.0244 (0.0010) N4 * -0.0289 (0.0010) C5 -0.1014 (0.0026) N1 0.0173 (0.0026) S2 Rms deviation of fitted atoms = 0.0201 3.4518 (0.0038) x + 1.0131 (0.0062) y + 14.7993 (0.0080) z = 13.9365 (0.0076) Angle to previous plane (with approximate esd) = 9.200 ( 0.107 ) * 0.0048 (0.0012) C21 * -0.0074 (0.0013) C22 * 0.0034 (0.0013) C23 * 0.0035 (0.0014) C24 * -0.0062 (0.0014) C25 * 0.0020 (0.0013) C26 0.0546 (0.0026) N1 0.0234 (0.0033) C27 Rms deviation of fitted atoms = 0.0049 3.6049 (0.0167) x + 0.8656 (0.1231) y + 14.5324 (0.0137) z = 13.6402 (0.0694) Angle to previous plane (with approximate esd) = 1.978 ( 0.397 ) * 0.0000 (0.0000) H01 * 0.0000 (0.0000) C5 * 0.0000 (0.0000) C21 0.0965 (0.0103) N1 Rms deviation of fitted atoms = 0.0000

### Fractional atomic coordinates and isotropic or equivalent isotropic displacement parameters (Å^2^)

**Table:**

	*x*	*y*	*z*	*U*_iso_*/*U*_eq_	
S1	0.68679 (6)	0.56520 (6)	0.74337 (2)	0.01368 (10)	
C2	0.7401 (3)	0.7619 (2)	0.69652 (9)	0.0141 (4)	
N3	0.6138 (2)	0.8948 (2)	0.71166 (8)	0.0164 (3)	
N4	0.4594 (2)	0.8505 (2)	0.76262 (8)	0.0164 (3)	
C5	0.4713 (3)	0.6807 (3)	0.78116 (9)	0.0141 (3)	
C11	1.0267 (2)	0.5674 (3)	0.61331 (9)	0.0129 (3)	
H11	0.908249	0.505970	0.585259	0.015*	
C12	1.2263 (3)	0.5825 (3)	0.56692 (9)	0.0125 (3)	
H12	1.336334	0.656908	0.593626	0.015*	
C13	1.3196 (3)	0.3958 (3)	0.55237 (9)	0.0132 (3)	
H13	1.225617	0.331025	0.516019	0.016*	
C14	1.3421 (3)	0.2844 (3)	0.62159 (9)	0.0129 (3)	
H14	1.457744	0.336867	0.653816	0.015*	
C15	1.1297 (3)	0.2862 (3)	0.66200 (9)	0.0135 (3)	
H15	1.014260	0.235018	0.629328	0.016*	
C16	1.1351 (3)	0.1833 (3)	0.73351 (10)	0.0159 (3)	
H16A	1.005890	0.214160	0.761185	0.019*	
H16B	1.129897	0.052814	0.722861	0.019*	
O1	1.07795 (19)	0.47115 (17)	0.67845 (7)	0.0135 (3)	
O2	1.1689 (2)	0.66863 (19)	0.50027 (7)	0.0153 (3)	
H02	1.264 (4)	0.740 (4)	0.4908 (15)	0.038 (8)*	
O3	1.52906 (19)	0.41758 (19)	0.52349 (7)	0.0168 (3)	
H03	1.522 (4)	0.482 (4)	0.4857 (13)	0.029 (7)*	
O4	1.3989 (2)	0.10451 (18)	0.60228 (7)	0.0162 (3)	
H04	1.471 (4)	0.067 (4)	0.6365 (12)	0.030 (7)*	
O5	1.3225 (2)	0.22073 (19)	0.77822 (7)	0.0178 (3)	
H05	1.382 (4)	0.122 (3)	0.7831 (14)	0.032 (8)*	
N1	0.3417 (2)	0.5861 (2)	0.82558 (8)	0.0156 (3)	
H01	0.353 (4)	0.473 (4)	0.8230 (13)	0.020 (6)*	
C21	0.1567 (3)	0.6454 (3)	0.86130 (9)	0.0154 (3)	
C22	0.0393 (3)	0.5129 (3)	0.89691 (10)	0.0191 (4)	
H22	0.082328	0.390608	0.894194	0.023*	
C23	−0.1396 (3)	0.5595 (3)	0.93618 (10)	0.0221 (4)	
H23	−0.216466	0.468512	0.960842	0.027*	
C24	−0.2085 (3)	0.7375 (3)	0.94008 (11)	0.0220 (4)	
C25	−0.0925 (3)	0.8667 (3)	0.90351 (11)	0.0230 (4)	
H25	−0.138366	0.988413	0.905038	0.028*	
C26	0.0901 (3)	0.8234 (3)	0.86446 (10)	0.0195 (4)	
H26	0.167780	0.914713	0.840330	0.023*	
S2	0.94984 (6)	0.79473 (6)	0.63645 (2)	0.01473 (10)	
C27	−0.4029 (3)	0.7886 (4)	0.98327 (13)	0.0322 (5)	
H27A	−0.533285	0.765916	0.953762	0.048*	
H27B	−0.395250	0.916469	0.996023	0.048*	
H27C	−0.406140	0.716487	1.028010	0.048*	

### Atomic displacement parameters (Å^2^)

**Table:**

	*U*^11^	*U*^22^	*U*^33^	*U*^12^	*U*^13^	*U*^23^
S1	0.01271 (18)	0.0111 (2)	0.01736 (19)	0.00212 (16)	0.00360 (13)	0.00076 (16)
C2	0.0134 (8)	0.0129 (10)	0.0161 (8)	0.0005 (6)	0.0007 (6)	−0.0002 (7)
N3	0.0159 (7)	0.0146 (8)	0.0188 (7)	0.0029 (6)	0.0043 (6)	0.0020 (6)
N4	0.0147 (7)	0.0164 (8)	0.0183 (7)	0.0024 (6)	0.0050 (6)	0.0016 (6)
C5	0.0127 (8)	0.0155 (9)	0.0140 (8)	0.0021 (7)	0.0004 (6)	−0.0013 (7)
C11	0.0123 (7)	0.0115 (8)	0.0149 (7)	0.0006 (7)	0.0020 (6)	0.0008 (7)
C12	0.0122 (7)	0.0120 (8)	0.0133 (7)	0.0005 (7)	0.0016 (5)	0.0015 (7)
C13	0.0114 (8)	0.0139 (9)	0.0145 (8)	0.0002 (6)	0.0031 (6)	−0.0006 (7)
C14	0.0141 (7)	0.0103 (8)	0.0145 (7)	0.0010 (7)	0.0031 (6)	0.0000 (7)
C15	0.0140 (7)	0.0103 (8)	0.0163 (7)	0.0011 (7)	0.0034 (6)	−0.0013 (7)
C16	0.0154 (8)	0.0153 (9)	0.0174 (8)	0.0007 (7)	0.0033 (6)	0.0017 (7)
O1	0.0160 (6)	0.0102 (6)	0.0144 (6)	0.0027 (5)	0.0040 (4)	0.0010 (5)
O2	0.0146 (6)	0.0151 (7)	0.0162 (6)	−0.0009 (5)	0.0017 (4)	0.0053 (5)
O3	0.0147 (6)	0.0166 (7)	0.0194 (6)	0.0030 (5)	0.0074 (5)	0.0049 (5)
O4	0.0202 (6)	0.0122 (7)	0.0163 (6)	0.0046 (5)	0.0039 (5)	0.0000 (5)
O5	0.0211 (7)	0.0135 (7)	0.0188 (6)	0.0027 (5)	−0.0010 (5)	0.0006 (5)
N1	0.0149 (7)	0.0127 (9)	0.0193 (7)	0.0012 (6)	0.0035 (5)	0.0009 (6)
C21	0.0120 (8)	0.0208 (10)	0.0136 (7)	0.0020 (7)	0.0007 (6)	−0.0009 (7)
C22	0.0177 (8)	0.0202 (10)	0.0195 (8)	0.0008 (7)	0.0013 (7)	0.0005 (7)
C23	0.0157 (8)	0.0301 (11)	0.0207 (8)	−0.0037 (9)	0.0038 (6)	0.0027 (9)
C24	0.0141 (8)	0.0309 (12)	0.0211 (9)	0.0000 (7)	0.0035 (7)	−0.0081 (8)
C25	0.0197 (9)	0.0225 (11)	0.0271 (10)	0.0042 (8)	0.0049 (8)	−0.0046 (8)
C26	0.0177 (8)	0.0194 (11)	0.0217 (8)	0.0012 (7)	0.0046 (7)	0.0001 (8)
S2	0.01492 (19)	0.0101 (2)	0.01939 (19)	0.00117 (15)	0.00557 (14)	0.00078 (16)
C27	0.0195 (9)	0.0386 (13)	0.0389 (11)	−0.0030 (10)	0.0112 (8)	−0.0133 (11)

### Geometric parameters (Å, º)

**Table:**

S1—C2	1.7322 (19)	C16—O5	1.438 (2)
S1—C5	1.7525 (18)	C16—H16A	0.9900
C2—N3	1.298 (2)	C16—H16B	0.9900
C2—S2	1.7473 (17)	O2—H02	0.82 (2)
N3—N4	1.397 (2)	O3—H03	0.84 (2)
N4—C5	1.309 (3)	O4—H04	0.81 (2)
C5—N1	1.358 (2)	O5—H05	0.83 (2)
C11—O1	1.421 (2)	N1—C21	1.411 (2)
C11—C12	1.529 (2)	N1—H01	0.84 (3)
C11—S2	1.811 (2)	C21—C26	1.389 (3)
C11—H11	1.0000	C21—C22	1.398 (3)
C12—O2	1.417 (2)	C22—C23	1.387 (3)
C12—C13	1.531 (3)	C22—H22	0.9500
C12—H12	1.0000	C23—C24	1.394 (3)
C13—O3	1.4311 (19)	C23—H23	0.9500
C13—C14	1.518 (2)	C24—C25	1.386 (3)
C13—H13	1.0000	C24—C27	1.513 (2)
C14—O4	1.431 (2)	C25—C26	1.397 (2)
C14—C15	1.534 (2)	C25—H25	0.9500
C14—H14	1.0000	C26—H26	0.9500
C15—O1	1.446 (2)	C27—H27A	0.9800
C15—C16	1.517 (2)	C27—H27B	0.9800
C15—H15	1.0000	C27—H27C	0.9800
			
C2—S1—C5	86.59 (9)	O5—C16—C15	113.27 (15)
N3—C2—S1	114.31 (13)	O5—C16—H16A	108.9
N3—C2—S2	119.74 (14)	C15—C16—H16A	108.9
S1—C2—S2	125.88 (10)	O5—C16—H16B	108.9
C2—N3—N4	113.29 (16)	C15—C16—H16B	108.9
C5—N4—N3	111.44 (15)	H16A—C16—H16B	107.7
N4—C5—N1	128.67 (17)	C11—O1—C15	110.51 (13)
N4—C5—S1	114.15 (14)	C12—O2—H02	108 (2)
N1—C5—S1	117.17 (15)	C13—O3—H03	109.9 (19)
O1—C11—C12	109.56 (13)	C14—O4—H04	106 (2)
O1—C11—S2	109.19 (11)	C16—O5—H05	104 (2)
C12—C11—S2	106.59 (13)	C5—N1—C21	128.45 (18)
O1—C11—H11	110.5	C5—N1—H01	115.6 (16)
C12—C11—H11	110.5	C21—N1—H01	113.9 (17)
S2—C11—H11	110.5	C26—C21—C22	119.40 (17)
O2—C12—C11	108.70 (13)	C26—C21—N1	124.47 (17)
O2—C12—C13	110.40 (13)	C22—C21—N1	116.11 (17)
C11—C12—C13	110.38 (15)	C23—C22—C21	120.26 (19)
O2—C12—H12	109.1	C23—C22—H22	119.9
C11—C12—H12	109.1	C21—C22—H22	119.9
C13—C12—H12	109.1	C22—C23—C24	121.12 (19)
O3—C13—C14	107.70 (13)	C22—C23—H23	119.4
O3—C13—C12	108.45 (14)	C24—C23—H23	119.4
C14—C13—C12	112.14 (14)	C25—C24—C23	117.89 (17)
O3—C13—H13	109.5	C25—C24—C27	120.9 (2)
C14—C13—H13	109.5	C23—C24—C27	121.2 (2)
C12—C13—H13	109.5	C24—C25—C26	122.0 (2)
O4—C14—C13	108.79 (13)	C24—C25—H25	119.0
O4—C14—C15	110.46 (15)	C26—C25—H25	119.0
C13—C14—C15	109.59 (13)	C21—C26—C25	119.31 (18)
O4—C14—H14	109.3	C21—C26—H26	120.3
C13—C14—H14	109.3	C25—C26—H26	120.3
C15—C14—H14	109.3	C2—S2—C11	102.94 (8)
O1—C15—C16	107.46 (13)	C24—C27—H27A	109.5
O1—C15—C14	107.97 (14)	C24—C27—H27B	109.5
C16—C15—C14	114.29 (14)	H27A—C27—H27B	109.5
O1—C15—H15	109.0	C24—C27—H27C	109.5
C16—C15—H15	109.0	H27A—C27—H27C	109.5
C14—C15—H15	109.0	H27B—C27—H27C	109.5
			
C5—S1—C2—N3	2.54 (14)	O1—C15—C16—O5	−74.18 (17)
C5—S1—C2—S2	179.52 (13)	C14—C15—C16—O5	45.6 (2)
S1—C2—N3—N4	−0.4 (2)	C12—C11—O1—C15	−66.50 (18)
S2—C2—N3—N4	−177.57 (12)	S2—C11—O1—C15	177.11 (10)
C2—N3—N4—C5	−3.0 (2)	C16—C15—O1—C11	−168.02 (13)
N3—N4—C5—N1	−175.82 (17)	C14—C15—O1—C11	68.24 (16)
N3—N4—C5—S1	4.95 (19)	N4—C5—N1—C21	2.2 (3)
C2—S1—C5—N4	−4.30 (14)	S1—C5—N1—C21	−178.54 (14)
C2—S1—C5—N1	176.37 (14)	C5—N1—C21—C26	−9.0 (3)
O1—C11—C12—O2	176.33 (14)	C5—N1—C21—C22	172.65 (16)
S2—C11—C12—O2	−65.64 (15)	C26—C21—C22—C23	−1.2 (3)
O1—C11—C12—C13	55.11 (18)	N1—C21—C22—C23	177.18 (16)
S2—C11—C12—C13	173.14 (11)	C21—C22—C23—C24	1.1 (3)
O2—C12—C13—O3	72.56 (16)	C22—C23—C24—C25	−0.1 (3)
C11—C12—C13—O3	−167.24 (13)	C22—C23—C24—C27	−179.64 (18)
O2—C12—C13—C14	−168.64 (13)	C23—C24—C25—C26	−0.9 (3)
C11—C12—C13—C14	−48.44 (18)	C27—C24—C25—C26	178.71 (19)
O3—C13—C14—O4	−69.37 (17)	C22—C21—C26—C25	0.3 (3)
C12—C13—C14—O4	171.39 (13)	N1—C21—C26—C25	−177.94 (17)
O3—C13—C14—C15	169.77 (14)	C24—C25—C26—C21	0.7 (3)
C12—C13—C14—C15	50.53 (19)	N3—C2—S2—C11	−164.51 (14)
O4—C14—C15—O1	−178.61 (13)	S1—C2—S2—C11	18.65 (13)
C13—C14—C15—O1	−58.76 (17)	O1—C11—S2—C2	−55.86 (12)
O4—C14—C15—C16	61.89 (19)	C12—C11—S2—C2	−174.12 (11)
C13—C14—C15—C16	−178.26 (16)		

### Hydrogen-bond geometry (Å, º)

**Table:**

*D*—H···*A*	*D*—H	H···*A*	*D*···*A*	*D*—H···*A*
O2—H02···O3^i^	0.82 (2)	1.87 (2)	2.6846 (19)	174 (3)
O3—H03···O4^i^	0.84 (2)	1.93 (2)	2.7375 (18)	161 (3)
O4—H04···N3^ii^	0.81 (2)	2.06 (2)	2.848 (2)	162 (3)
O5—H05···N4^ii^	0.83 (2)	2.11 (2)	2.899 (2)	159 (3)
O5—H05···N3^ii^	0.83 (2)	2.60 (2)	3.282 (2)	141 (2)
N1—H01···O5^iii^	0.84 (3)	2.05 (3)	2.853 (2)	158 (2)
C14—H14···S1^iv^	1.00	2.74	3.7044 (18)	163
C15—H15···O2^v^	1.00	2.66	3.577 (2)	153
C11—H11···O3^iii^	1.00	2.68	3.652 (2)	164
C16—H16*B*···S2^vi^	0.99	2.71	3.570 (2)	145

Symmetry codes: (i) −*x*+3, *y*+1/2, −*z*+1; (ii) *x*+1, *y*−1, *z*; (iii) *x*−1, *y*, *z*; (iv) *x*+1, *y*, *z*; (v) −*x*+2, *y*−1/2, −*z*+1; (vi) *x*, *y*−1, *z*.
